# Molecular Effects of Chronic Exposure to Palmitate in Intestinal Organoids: A New Model to Study Obesity and Diabetes

**DOI:** 10.3390/ijms23147751

**Published:** 2022-07-13

**Authors:** Agnese Filippello, Stefania Di Mauro, Alessandra Scamporrino, Sebastiano Alfio Torrisi, Gian Marco Leggio, Antonino Di Pino, Roberto Scicali, Maurizio Di Marco, Roberta Malaguarnera, Francesco Purrello, Salvatore Piro

**Affiliations:** 1 Department of Clinical and Experimental Medicine, Internal Medicine, Garibaldi-Nesima Hospital, University of Catania, 95122 Catania, Italy; agnese.filippello@gmail.com (A.F.); 8stefaniadimauro6@gmail.com (S.D.M.); alessandraska@hotmail.com (A.S.); antonino.dipino@unict.it (A.D.P.); robertoscicali@gmail.com (R.S.); maurizio.dimarco@studium.unict.it (M.D.M.); salvatore.piro@unict.it (S.P.); 2 Department of Biomedical and Biotechnological Sciences, University of Catania, Via S. Sofia, 64, 95123 Catania, Italy; sebastiano.torrisi@unict.it (S.A.T.); gianmarco.leggio@unict.it (G.M.L.); 3 Faculty of Medicine and Surgery, “Kore” University of Enna, 94100 Enna, Italy; roberta.malaguarnera@unikore.it

**Keywords:** intestinal cells, lipotoxicity, intestinal organoids

## Abstract

Intestinal cell dysfunctions involved in obesity and associated diabetes could be correlated with impaired intestinal cell development. To date, the molecular mechanisms underlying these dysfunctions have been poorly investigated because of the lack of a good model for studying obesity. The main aim of this study was to investigate the effects of lipotoxicity on intestinal cell differentiation in small intestinal organoid platforms, which are used to analyze the regulation of cell differentiation. Mouse intestinal organoids were grown in the presence/absence of high palmitate concentrations (0.5 mM) for 48 h to simulate lipotoxicity. Palmitate treatment altered the expression of markers involved in the differentiation of enterocytes and goblet cells in the early (Hes1) and late (Muc2) phases of their development, respectively, and it modified enterocytes and goblet cell numbers. Furthermore, the expression of enteroendocrine cell progenitors (Ngn3) and I cells (CCK) markers was also impaired, as well as CCK-positive cell numbers and CCK secretion. Our data indicate, for the first time, that lipotoxicity simultaneously influences the differentiation of specific intestinal cell types in the gut: enterocytes, goblet cells and CCK cells. Through this study, we identified novel targets associated with molecular mechanisms affected by lipotoxicity that could be important for obesity and diabetes therapy.

## 1. Introduction

The onset of obesity and associated type 2 diabetes mellitus (T2DM) is related to the increase in plasmatic free fatty acids (FFAs) [1]. The increased levels of FFAs contribute to insulin resistance, the inflammation of many tissues, and pancreatic islet cell dysfunction [2]. During the past few years, it has been reported that chronic elevation of circulating FFAs is able to cause an imbalance in gut homeostasis and intestinal epithelial dysfunction [3,4].

Keeping intestinal tract physiological function is essential for the prevention and treatment of several metabolic disorders determined by chronic hyperlipidemia [5]. Although it is amply reported that metabolic diseases are associated with intestinal cell dysfunction [6,7,8] and despite diet having a deep impact on gut physiology, the molecular mechanisms of these relationships are still poorly understood because of the lack of a good model for studying obesity in the intestine.

The intestinal epithelium is subjected to renewal every 4–5 days and is composed of terminally differentiated cells. The differentiation of intestinal cells is a continuous process strictly regulated in the gut; these cells arise from intestinal stem cells (ISCs) expressing leucine-rich repeats containing G-protein coupled receptor 5 (Lgr5) located in intestinal crypts that give rise to proliferating transit-amplifying cells (TA). Each of these cells can generate absorptive enterocytes and all secretory cell types such as goblet cells, Paneth cells, enteroendocrine cells (EECs) and tuft cells through the regulated expression of specific transcription factors (TFs). Hairy and enhancer of split-1 (Hes1) transcription factor is required for differentiation into absorptive enterocytes, while protein atonal homolog 1 (Atoh1) expression is involved in the determination of secretory cell fate. Kruppel-like factor 4 (Klf4) and Sox9 are required for goblet cell and Paneth cell differentiation, respectively. EECs require the transient expression of Neurogenin3 (Neurog3), while the development of Tuft cells requires the expression of the Pou domain, class 2, transcription factor 3 (Pou2f3) [9,10].

It has been recently reported that the expression of TFs controlling intestinal epithelial cell differentiation is deregulated in metabolic diseases [11,12,13], and it has been hypothesized that diet changes can alter intestinal cell differentiation and contribute to their dysfunction and deregulation. In the last few years, intestinal organoids have been used to study the development of intestinal cells [10]. Organoids contain multiple gut-specific cell types that self-organize in three dimensions (3D) and show organ-specific structure and function [14]. Organoids have recently started to be used in metabolic disease research [15]; our previous finding demonstrated that chronic hyperglycemia treatment impaired enteroendocrine L cell differentiation and decreased stem cell proliferative capacity in intestinal organoids [16].

Among the dietary products, as well as glucose, palmitic acid has well-known lipotoxic effects on many human organs [17,18,19]. At the intestinal level, palmitate is known to impair gut insulin sensitivity and is correlated to an enhanced incidence of obesity and T2DM [2,20,21]. Furthermore, it has been reported that chronic exposure to palmitate impaired cell differentiation in several tissues [22,23,24]; however, this effect is poorly studied in intestinal cells.

It is known that chronic hyperglycemia and hypernefemia act both in a synergic [25] and distinct manner [26,27] at the cellular level. Since intestinal cell differentiation is strictly regulated by several TFs, glucose or palmitate can have a different effect on the expression of these TFs. Thus, our model allows for the isolation of the specific in vitro effect of free fatty acids on intestinal cell differentiation. Moreover, to the best of our knowledge, the specific molecular mechanisms explaining the pathophysiology of gut epithelial cell differentiation and the role of lipotoxicity in the modulation of the expression of regulatory networks involved in the identity and differentiation of intestinal epithelial cells have not been clearly established.

The main aim of this study was to test if chronic palmitate treatment (lipotoxicity) causes dysfunction of epithelial intestinal cell differentiation, leading to impaired development and consequent alteration of intestinal cell number end/or function. The dysfunction of these cells could result in impaired gut physiology observed in obesity. To analyze the effect of lipotoxicity, considered as cellular dysfunction before cell death induction, on the differentiation of intestinal cells, we used small intestinal organoid platforms. In this study, the gene expression of TFs modulating identity and differentiation of intestinal cells and the gene expression of mature cell markers were analyzed by using intestinal organoids chronically treated with palmitate. Furthermore, the cellular localization of differentially expressed genes and their secretion products were evaluated.

## 2. Results

### 2.1. Effect of Palmitate Treatment on Organoid Viability

To evaluate lipotoxicity induced by palmitate treatment, we performed the MTT assay in organoids exposed for different periods (24, 48, or 72 h) to increasing palmitate concentrations (0.25, 0.5 and 1 mM).

**Figure 1:**
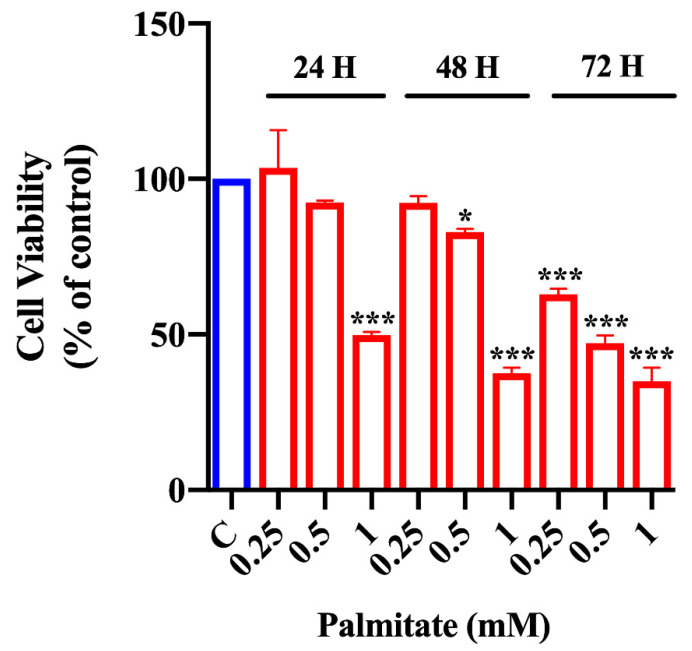
Effect of palmitate on cell viability in intestinal organoids. MTT assay in intestinal organoids exposed to increasing concentrations of palmitate (0.25, 0.5 and 1 mM) after 24, 48, and 72 h (*n* = 6). Data are expressed as means ± SD of 570 nM absorbance to % of controls. One-way ANOVA followed by Bonferroni test: * *p* < 0.05, *** *p* < 0.001 compared to controls.

As shown in Figure 1, only the highest palmitate dose (1 mM) exerted a strong cytotoxic effect at 24 h. At 48 h of treatment, 0.25 mM palmitate did not alter cell viability, while 0.5 mM palmitate induced a minimal but significant reduction of cell viability with respect to control organoids. On the contrary, palmitate treatment for 72 h was toxic versus control organoids at all the analyzed concentrations. Statistical *p* values are reported in Table S1. Since 0.5 mM palmitate treatment for 48 h represented the lipotoxicity condition (cellular dysfunction before cell death pathway activation) better than the other observed concentrations, we chose this dose–time combination for subsequent experiments.

### 2.2. Effect of Palmitate Exposure on Morphology and Stemness Features of Organoids

**Figure 2:**
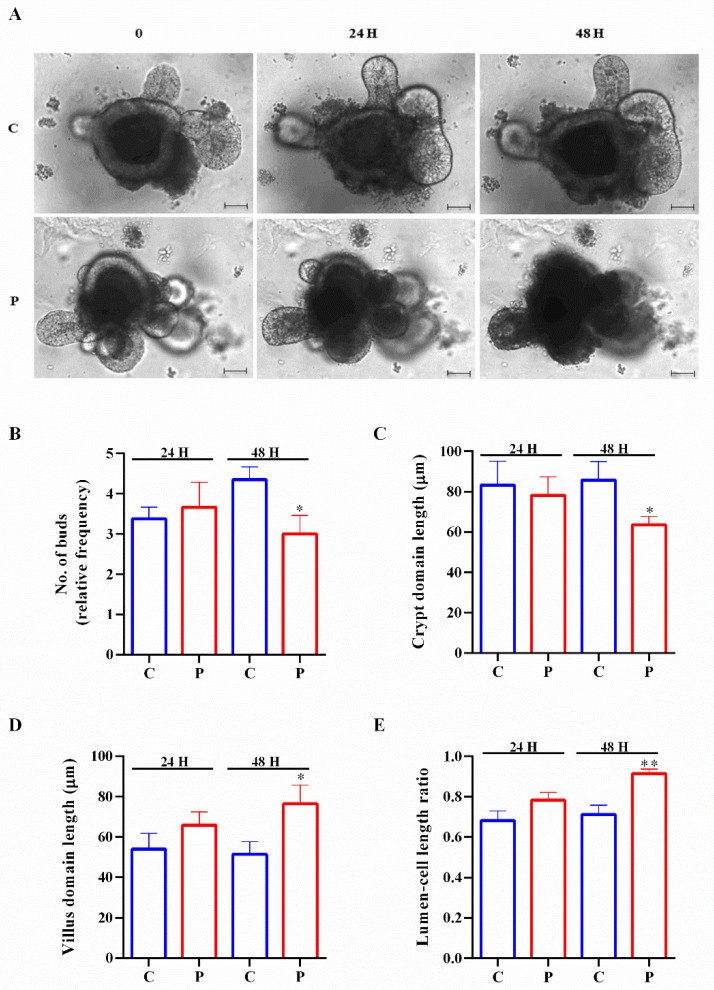
Lipotoxicity affects intestinal organoid growth. (**A**) Representative brightfield images of intestinal organoids following 0.5 mM palmitate exposure for 24 and 48 h. Scale bar, 50 μm. Number of buds (**B**), crypt domain length (**C**), villus domain length (**D**) and lumen–cell length ratio (**E**) in controls and treated organoids (*n* = 6). Data are expressed as means ± SD. Unpaired Student’s *t* test: * *p* < 0.05, ** *p* < 0.01. C, control; P, palmitate.

In order to analyze if palmitate treatment affected the growth of organoids, we compared the principal morphological characteristics of intestinal organoids treated with palmitate (0.5 mM) for different periods (24 and 48 h) compared to controls. The two types of organoids had differences in their general appearance during growth (Figure 2A). Organoids exposed to palmitate for 48 h showed a reducing trend in the proportion of budded palmitate-treated organoids (those with >3 buds) with respect to control organoids and a slightly decreased crypt domain length (Figure 2B,C). On the contrary, the villus domain length and the ratio between the lumen and cell length were increased after 48 h of exposure (Figure 2D,E). Statistical *p* values of organoid morphological features are shown in Table S2.

**Figure 3:**
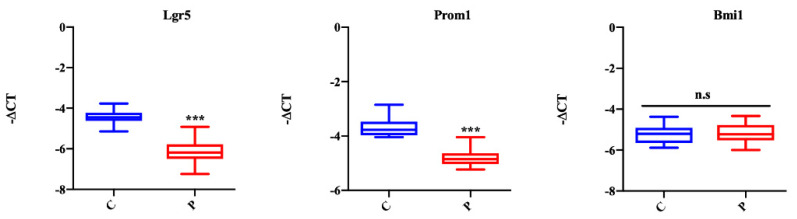
Influence of palmitate treatment on intestinal organoid proliferation. Lgr5, stem cells; Prom1, TA cells; Bmi1, +4 cells. Data generated from three biological replicates from five mice. Unpaired Student’s *t* test: *** *p* < 0.001; n.s. not significant. C, control; P, palmitate.

To analyze the effect of palmitate on organoid stemness features, we studied mRNA expression of the Lgr5 stem cell marker, the prominin 1 (Prom1) TA cell marker and the polycomb ring finger oncogene (Bmi1), a marker for reserve intestinal stem cells. According to the growth data (reduced number of buds and crypt domain length), the expression of stem cell and TA cell markers was significantly decreased in organoids treated with palmitate, while Bmi1 was not dysregulated between the two groups (Figure 3). These results suggest a reduction of stem cell proliferative capacity of organoids exposed to palmitate.

### 2.3. Effect of Lipotoxicity on Intestinal Cell Differentiation

**Figure 4:**
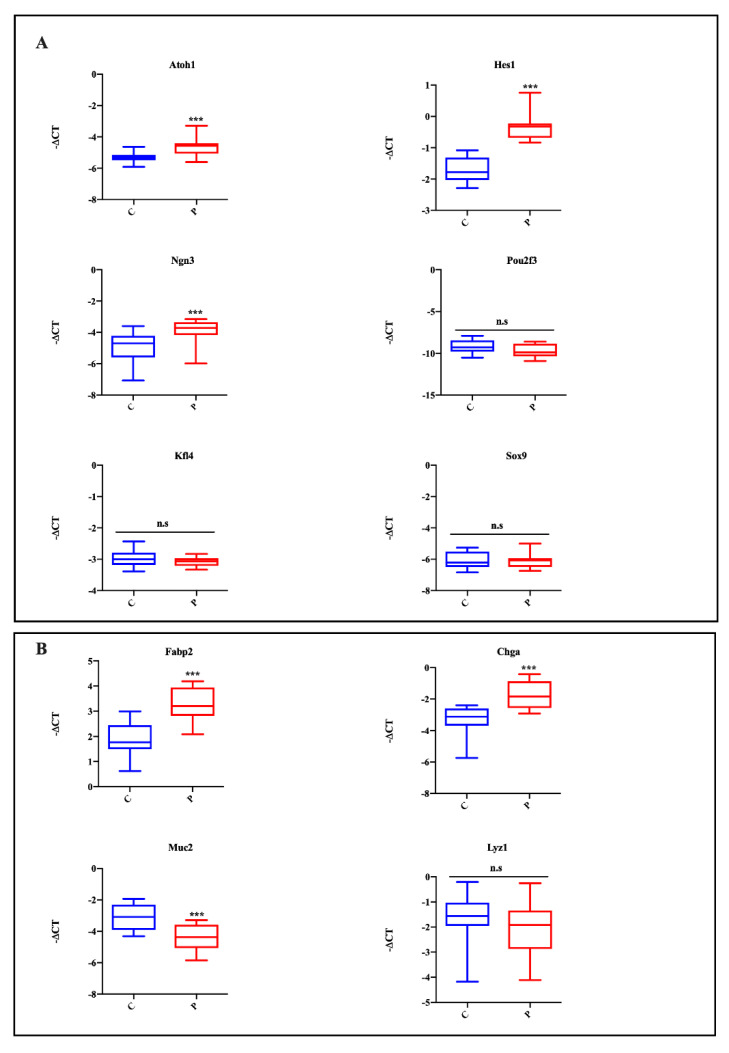
Effect of palmitate treatment TFs and specific intestinal epithelial lineage marker expression. Atoh1, Hes1, Neurog3, Pou2f3, Klf4 and Sox9 (**A**); Fabp2, ChgA, Muc2 and Lyz1 (**B**). Data generated from three biological replicates from five mice. Unpaired Student’s *t* test: *** *p* < 0.001; n.s., not significant. C, control; P, palmitate.

To study if palmitate affects organoid differentiation, we initially analyzed the expression of TFs regulating intestinal cell differentiation. As shown in Figure 4A, murine small intestinal organoids treated with palmitate showed an increased expression of Atoh1 and Hes1, which are TFs important for secretory and absorptive cell lineages, respectively. Similarly to the Atoh1 expression profile, the expression of Ngn3, a TF that early identifies endocrine progenitor cells, was increased in the palmitate-exposed group with respect to controls (Figure 4A). However, the expression of the tuft cell marker (Pou2f3), the goblet cell marker (Klf4) and Paneth cell marker SRY (Sox9) were unaffected in the controls and the palmitate-treated group (Figure 4A).

To assay the effect of lipotoxicity-induced impaired expression of TFs (Atoh1, Hes1 and Ngn3) on intestinal epithelial lineages, the expression of individual cell type markers was analyzed. According to TF data expression, the mRNA levels of the enterocyte marker, fatty acid binding protein 2, intestinal (Fabp2) and the EEC marker, chromogranin A (ChgA), were significantly increased in the palmitate-treated group (Figure 4B). Instead, the goblet cell marker, mucin 2 (Muc2), was significantly decreased in organoids exposed to palmitate. Finally, the expression of the Paneth cell marker, lysozyme 1 (Lyz1), was unaltered between controls and the palmitate-treated group (Figure 4B).

**Figure 5:**
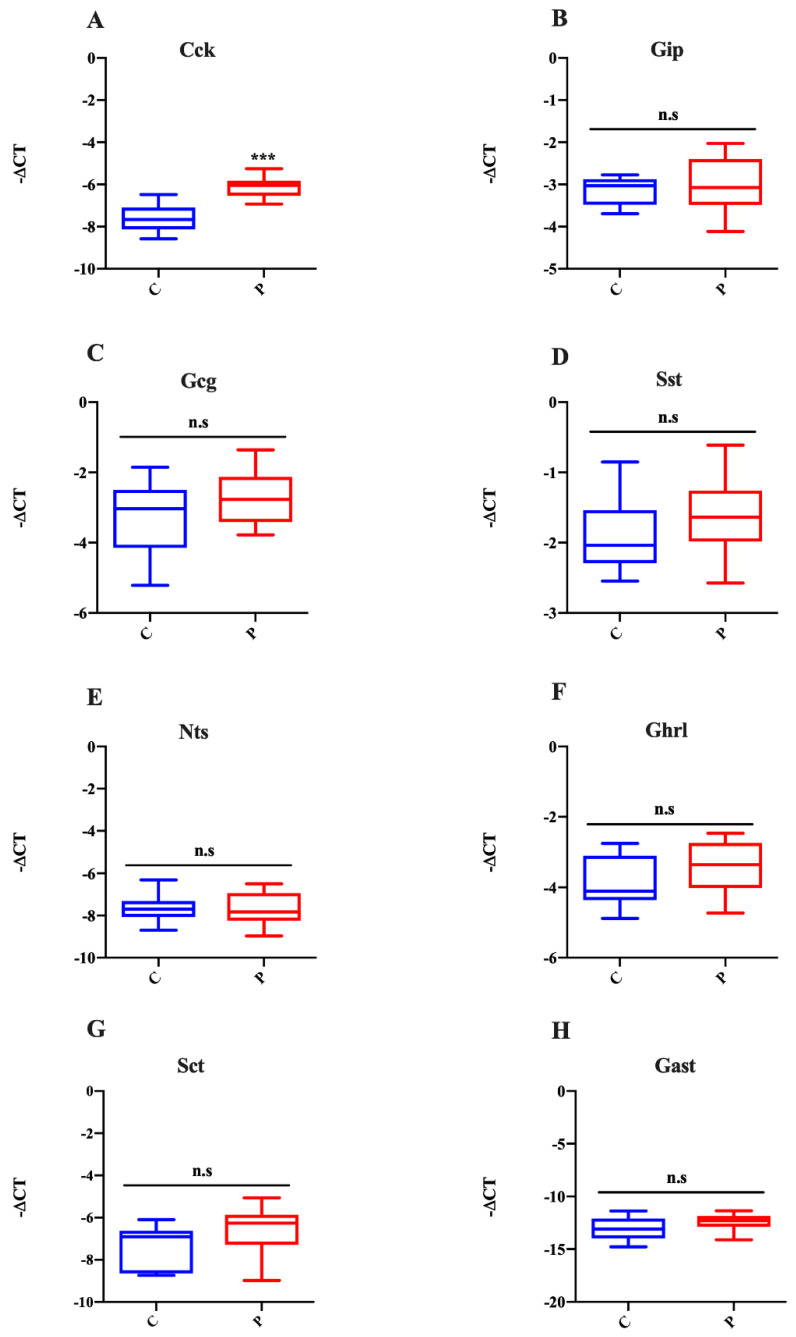
Action of palmitate exposure on EEC markers. Expression of CCK (**A**), Gip (**B**), Glp-1 (**C**), Sst (**D**), Nts (**E**), Ghrl (**F**), Sct (**G**) and Gast (**H**) in intestinal organoids treated with palmitate for 48 h. Data generated from three biological replicates from five mice. Unpaired Student’s *t* test: *** *p* < 0.001; n.s., not significant. C, control; P, palmitate.

Since ChgA is a general marker of EECs, to evaluate the effect of its increased expression, we analyzed the mRNA expression of the most important gastrointestinal hormones that characterize different types of EECs in organoids treated with palmitate. In accordance with Ngn3 and ChgA expression, the I cell marker (Cck) was significantly upregulated in the palmitate-treated group (Figure 5A), while the mRNA expression levels of the markers for K cells (Gip), L cells (Gcg), D cells (Sst), N-cells (Nts), X cells (Ghrl), S cells (Sct), and G cells (Gast) were unaffected by lipotoxicity (Figure 5B–H). Statistical analysis of all examined transcript levels is reported in Table S3.

### 2.4. Palmitate Treatment Impairs Intestinal Cell Fate in Organoids

**Figure 6:**
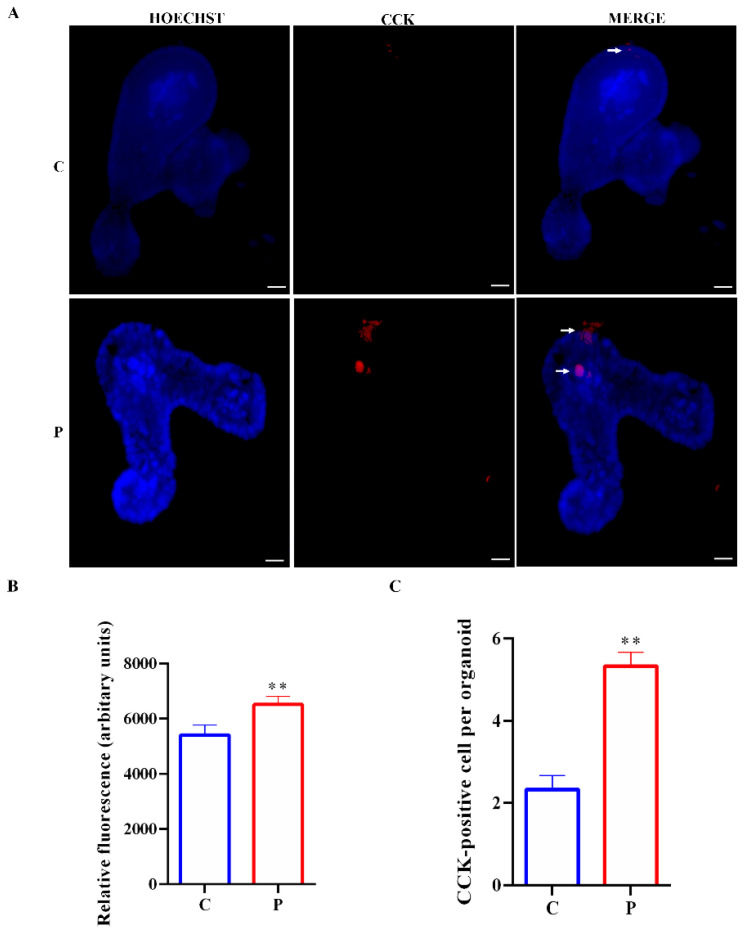
Expression of CCK in small intestinal organoids. (**A**) Representative images of controls and organoids treated with palmitate for 48 h. CCK cells, inside organoids, are labeled in red by CCK expression (white arrows). Nuclei are labeled by Hoechst. Scale bar, 50 μm. (**B**) Quantification of the CCK fluorescence intensity. (**C**) CCK cell numbers in control organoids and organoids treated with palmitate. (*n* = 6). Data are expressed as means ± SD. Unpaired Student’s *t* test: ** *p* < 0.01. C, control; P, palmitate.

To investigate if lipotoxicity can change the number and localization of I cells, goblet cells and enterocytes, immunostainings for intracellular CCK, MUC2 and FABP2 were performed in organoids treated with palmitate. As shown in Figure 6A,B, the expression of CCK was remarkably enhanced in palmitate organoids with respect to controls. Moreover, CCK organoid localization was altered in organoids exposed to palmitate; CCK was also localized in the lumen of organoids. Finally, in agreement with the increased gene expression of Atoh1, Ngn3 and CCK, CCK cell numbers were increased in palmitate-exposed intestinal organoids compared with controls (Figure 6C).

**Figure 7:**
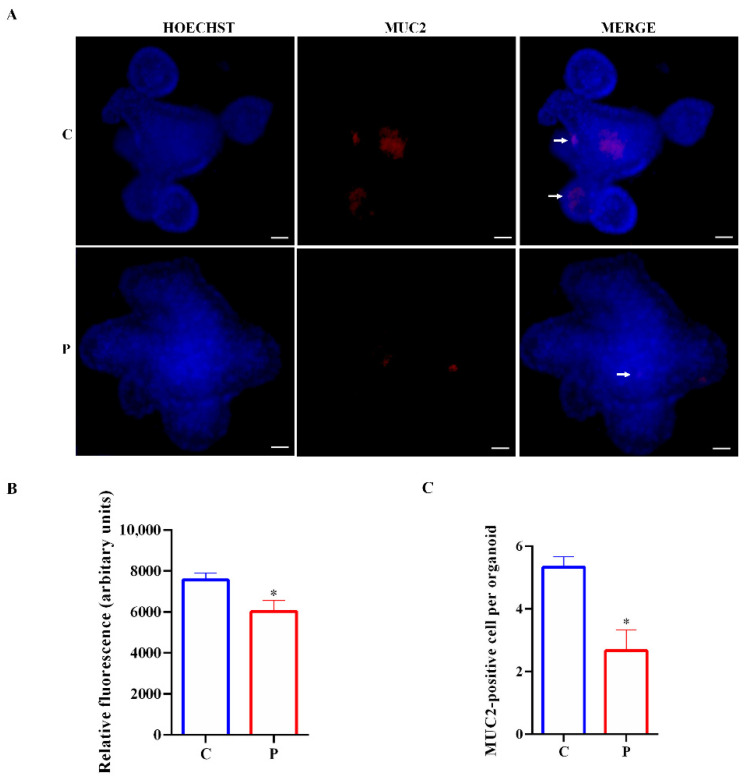
Expression of MUC2 in small intestinal organoids. (**A**) Representative images of controls and organoids treated with palmitate for 48 h. Goblet cells, inside organoids, are labeled in red by MUC2 expression (white arrows). Nuclei are labeled by Hoechst. Scale bar, 50 μm. (**B**) Quantification of the MUC2 fluorescence intensity. (**C**) MUC2 cell numbers in control organoids and organoids treated with palmitate. (*n* = 6). Data are expressed as means ± SD. Unpaired Student’s *t* test: * *p* < 0.05. C, control; P, palmitate.

Furthermore, in agreement with MUC2 downregulation in organoids exposed to palmitate, we also observed a reduction of MUC2-positive cells (Figure 7A–C).

**Figure 8:**
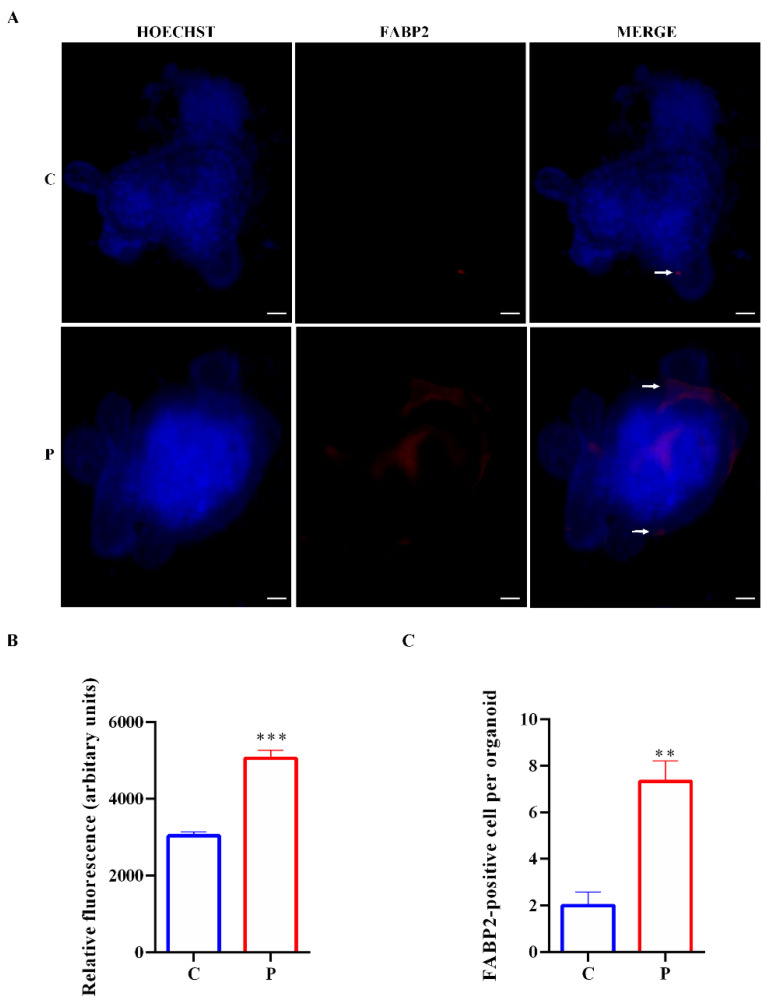
Expression of FABP2 in intestinal organoids. (**A**) Representative images of controls and organoids treated with palmitate for 48 h. Enterocytes, inside organoids, are labeled in red by FABP2 expression (white arrows). Nuclei are labeled by Hoechst. Scale bar, 50 μm. (**B**) Quantification of the FABP2 fluorescence intensity. (**C**) FABP2 cell numbers in control organoids and organoids treated with palmitate. (*n* = 6). Data are expressed as means ± SD. Unpaired Student’s *t* test: ** *p* < 0.01, *** *p* < 0.001. C, control; P, palmitate.

The intensity of FABP2 expression was significantly augmented in palmitate organoids compared to untreated controls, and FABP2 was also localized in the lumen of organoids treated with palmitate (Figure 8A,B). The FABP2 cell numbers were increased in organoids exposed to palmitate with respect to controls, consistent with the increased expression of Hes1 and Fabp2 (Figure 8C).

These data demonstrate that lipotoxicity affects mature intestinal cell formation.

**Figure 9:**
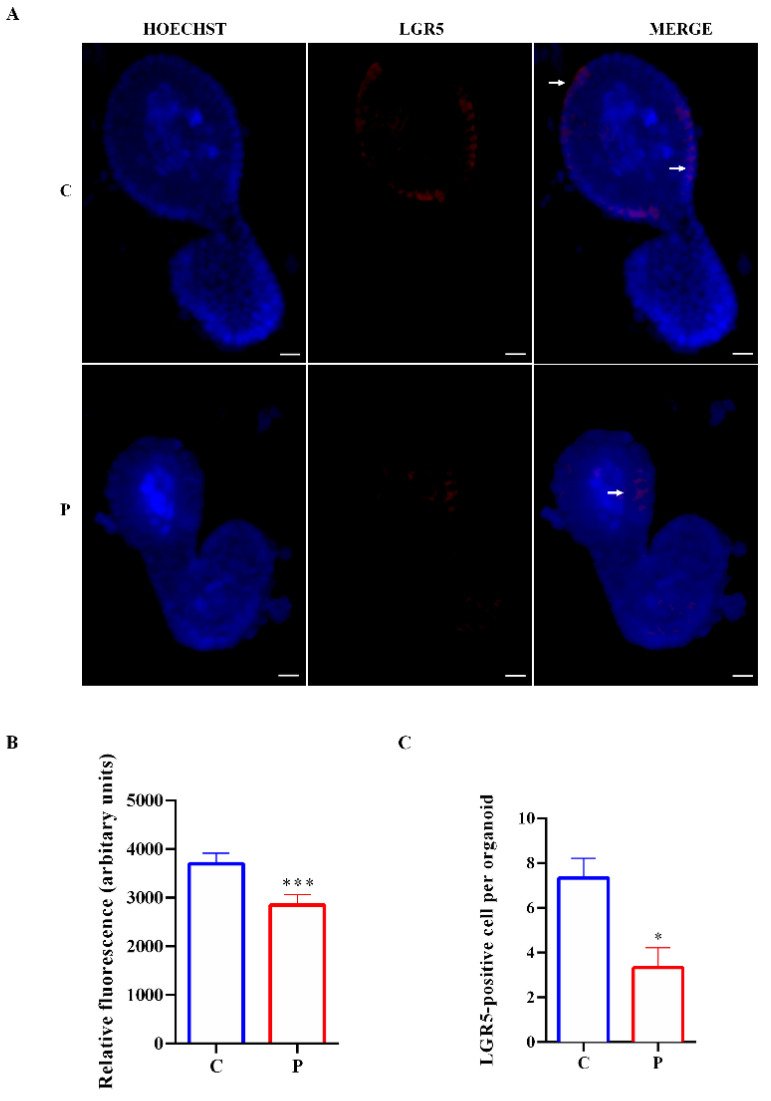
Expression of LGR5 in intestinal organoids. (**A**) Representative images of controls and organoids treated with palmitate for 48 h. Intestinal stem cells, inside organoids, are labeled in red by LGR5 expression (white arrows). Nuclei are labeled by Hoechst. Scale bar, 50 μm. (**B**) Quantification of the LGR5 fluorescence intensity. (**C**) LGR5 cell numbers in control organoids and organoids treated with palmitate. (*n* = 6). Data are expressed as means ± SD. Unpaired Student’s *t* test: * *p* < 0.05, *** *p* < 0.001. C, control; P, palmitate.

Finally, according to organoid growth and Lgr5 transcript expression data, we found that chronic exposure to palmitate affects the development of organoids. To evaluate LGR5-positive cell expression, we performed immunofluorescence experiments for LGR5. The expression of LGR5 and LGR5 cell numbers was strongly reduced in palmitate-exposed organoids compared with controls (Figure 9A–C). Table S4 reports the *p* values of the immunofluorescence data.

### 2.5. Effects of Palmitate Treatment on MUC2 and CCK Production in Intestinal Organoids

**Figure 10:**
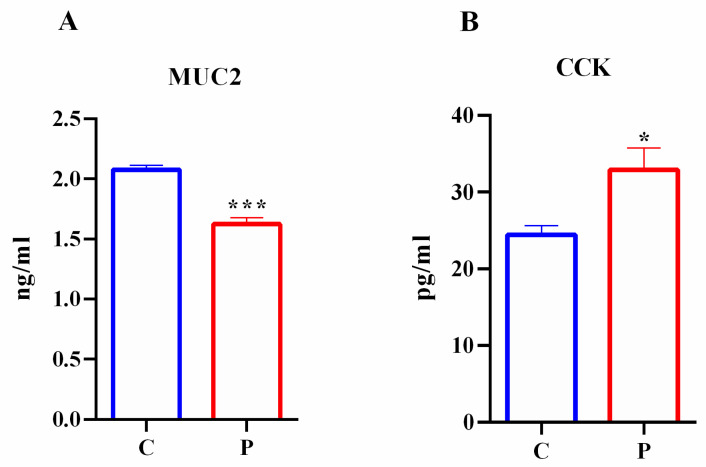
Effects of palmitate exposure on MUC2 and CCK production in intestinal organoids. MUC2 (**A**) and CCK (**B**) secretion from intestinal organoids treated with palmitate. (*n* = 3). Data are expressed as means ± SD. Unpaired Student’s *t* test: * *p* < 0.05, *** *p* < 0.001. C, control; P, palmitate.

Goblet cells and I cells produced MUC2 and CCK, respectively, which are important for gut protection and physiology. To analyze if lipotoxicity was able to affect goblet cell and I cell functional activity, MUC2 and CCK secretion were performed in intestinal organoids exposed to palmitate. According to previous data, in the culture medium of intestinal organoids treated with palmitate, there was a reduction in the mucous glycoprotein MUC2 level (Figure 10A). These data demonstrated that palmitate impaired not only Muc2 expression and localization but also MUC2 secretion by the intestinal organoids.

However, in agreement with CCK data expression, the level of CCK was significantly increased in the culture medium of intestinal organoids exposed to palmitate compared to control (Figure 10B). These results indicated that palmitate exposure altered not the only the expression of TFs (Atoh1 and Ngn3) regulating CCK expression, but also the functional activity of I cells as shown by impaired CCK secretion in intestinal organoids. Table S5 shows the *p* values of MUC2 and CCK secretion.

## 3. Discussion

Our study provides a snapshot of the chronic hyperlipidemia effect on intestinal cell differentiation by using small intestinal organoid platforms. Organoids allow for the investigation of how biologically active intestinal cells interact in the gut, how TFs regulating gut cell differentiation interact with each other, and how nutritional changes causing metabolic diseases could impair these aspects [16,28,29]. In addition, our 3D platform could represent a starting point for the development of on-chip model in order to obtain an enhancement of the understanding of physiopathological alterations that could lead to an improvement in the prevention and treatment of complex chronic diseases such as obesity and diabetes.

Although palmitic acid, a well-known inductor of nutritional changes in metabolic diseases, causes severe intestinal dysfunction, to date, the effect of chronic palmitate treatment on intestinal cell differentiation has not been investigated because of the lack of a good model for studying obesity. Moreover, it is not known whether the dysfunctions of intestinal cells concern the early or final stage of their development. For this purpose, by simulating lipotoxicity in vitro, we analyzed the expression of regulatory networks regulating intestinal cell identity and differentiation and their functional markers on intestinal organoids.

**Figure 11:**
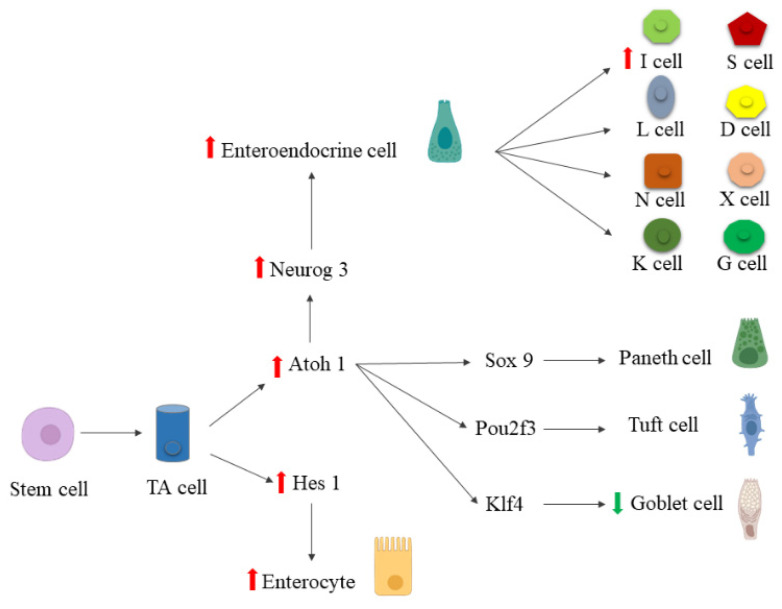
Schematic representation of palmitate effect on the expression of TFs regulating intestinal cell differentiation. Red arrows represent upregulated transcripts. The green arrow represents the downregulated transcript.

In this study, we demonstrate that chronic treatment with palmitate (0.5 mM palmitate for 48 h) impairs intestinal cell differentiation at different stages of their development. Lipotoxicity alters the differentiation of enterocytes and goblet cells in the early and late phases of their development, respectively. Furthermore, for the first time, we provide evidence that chronic exposure to palmitate impairs TF expression, Atoh1 and Ngn3, associated with the differentiation of early and late endocrine progenitors, respectively. More in detail, the dysregulation of these TFs seems to be associated with increased I (CCK) cell endocrine specification in our experimental model (Figure 11).

In current years, the molecular mechanisms of the relationship between lipotoxicity and dysfunction of intestinal cells (enterocytes, goblet cells and EECs) have been principally demonstrated only at the level of single-cell type in an immortalized intestinal cell line or primary culture [4,20,30]. These mechanisms have not been elucidated in intestinal organoids that contain all specialized intestinal epithelial cells and their precursors. First, we showed that chronic exposure to palmitate increased FABP-2 positive cells in intestinal organoids. FABP-2 is expressed in small intestinal enterocytes, which facilitates the transport of FFAs contributing to lipid absorption and metabolism [31]. Several studies have reported that polymorphisms of FABP2 are correlated with an increase in obesity, TD2M and metabolic syndrome onset [32,33,34]. The role of FABP2 in metabolic diseases was also demonstrated in Fabp2-null mice that showed a significant reduction in FFA incorporation into triglycerides [35]. Kaufman et al. recently reported that rats after gastric bypass surgery showed a decreased triglyceride secretion by enterocytes due to reduced FABP2 expression and enterocyte number compared to obese rats [36]. These findings indicated that the increased expression of FABP2 in our experimental model could represent a new effect of an obesogenic diet on enterocyte function in intestinal organoids. In our study, we also observed an increase in villus domain length and an altered pattern of FABP-2 cell localization in intestinal organoids exposed to palmitate according to previous data observed in villi and enterocytes isolated from obesity and prediabetes mice [4]. Moreover, we showed that for the first time in intestinal organoids, lipotoxicity-induced enterocyte dysfunction seems to occur at an early stage of their development at the level of enterocyte precursors as shown by increased Hes1 expression, a TF essential for regulating enterocyte differentiation.

In the intestinal epithelium, goblet cells are involved in synthesizing and secreting mucus to form a gut barrier that protects the epithelium against microbes and luminal substances [37]. In the absence of mucin (MUC2), bacteria are in contact with the intestinal epithelium, and they induce inflammation that is observed in several diseases [38]. Everard et al. have shown that the gut barrier could be impaired by diets rich in saturated fatty acids that induce epithelium leakage [39]. Moreover, the mutations of the Muc2 gene can trigger stress on the endoplasmic reticulum and increase levels of inflammatory cytokines as observed in obese patients [40,41]. Nevertheless, despite the strong association between goblet cells and obesity, an extensive molecular characterization of these cells in the gut has never been performed. In our findings, we showed for the first time that lipotoxicity reduced the number of goblet cells; thus, the production and secretion of MUC2 were significantly decreased in organoids. Our results are consistent with a previous report showing reduced MUC2 secretion in the human colon LS 174T cell line exposed to palmitate. In these cells, altered MUC2 secretion induced stress of the endoplasmic reticulum-associated with mucin misfolding [3]. Finally, we demonstrated that in our experimental model, lipotoxicity did not affect the expression of goblet cell differentiation transcription factor (Kfl4); therefore, palmitate seems to impair only mature goblet cells.

Among EECs, I cells secrete the hormone CCK in response to fat and protein [42]. CCK has many physiological actions such as regulating digestion and satiety, inhibiting gastric acid secretion and stimulating insulin secretion [43]. Whether obesity and TD2M affect CCK secretion is controversial because of the lack of a good experimental model to study I cells secreting CCK. In some studies, serum CCK levels were decreased in obese subjects [44], but not in others [45,46]. In our study, we showed that in intestinal organoids exposed to palmitate, the mRNA level of CCK and positive CCK cell number are significantly augmented. Consequently, secretion of CCK is also increased according to CCK increased release observed in obese patients [47]. The effect of palmitate appears to act during the early stages of I cell development, as demonstrated by the increased expression of Atoh1 and Ngn3 in our experimental model. The consequences of increased CCK production could be different. Weickert et al. reported the correlation between hyperinsulinemia and increased levels of plasma CCK [48]. Since supraphysiological doses of CCK stimulate pancreatic insulin secretion, higher levels of circulating CCK induced by chronic palmitate treatment could be a compensatory response to insulin resistance in the prediabetic condition, which leads to hyperinsulinemia. Moreover, it is reported that CCK stimulates contraction of the gallbladder, thereby markedly increasing the delivery of bile acids into the duodenum [49]. Lund et al. showed the correlation between increased GLP-1 secretion and bile acids in human intestinal organoids [50]; therefore, it could be hypothesized that CCK indirectly regulates GLP-1 secretion. Further studies to demonstrate these hypotheses will be needed. Moreover, it has been demonstrated that bile acids promoted ISC proliferation [51]; therefore, released CCK might have a compensatory effect on increasing the impaired LGR-5 expression induced by the chronic exposure to palmitate observed in our experimental model. Finally, regarding LGR5 expression, several studies reported that exogenous and endogenous factors, including caloric restriction, fasting, and glucose, affect the homeostasis of ISCs [16,52,53], and the reduction of LGR5 also appears to be related with a switch of differentiation of specific cell types, as previously reported [54,55]. Therefore, the palmitate-induced decreased LGR5 expression observed in our experimental model could be related to the increased differentiation of enterocytes and secretory cells in intestinal organoids.

The results of this study indicated that chronic exposure to palmitate affects enterocytes and secretory cell differentiation at different stages of their development, impairing specific TF expression in mouse intestinal organoids. These cells are important for gastrointestinal endocrine secretion. Since gastrointestinal hormones play a pivotal role in obesity and diabetes pathogenesis, these alterations could be interesting for the understanding of metabolic disease pathogenesis. In our study, we evaluated for the first time the effects of palmitate on all types of intestinal cells simultaneously trying to understand if the dysfunction of one cell type can influence the development of another cell type. In summary, these data add novel evidence that can lead to a better understanding of the cell intestinal alterations in the context of obesity and associated diabetes.

In conclusion, our findings provide a new molecular study model for obesity using a small intestinal organoid platform. This study identified new targets involved in novel molecular signaling pathways impaired by lipotoxicity that could be useful for better treatment of obesity and diabetes.

## 4. Materials and Methods

### 4.1. Animals

Intestinal organoids were obtained from male C57BL/6J mice (Charles River Laboratories Italia, Italy, 8–12 weeks old). Animals were group-housed with free access to water and chow in an air-conditioned room, with a 12 h light-dark cycle and under stable temperature (23 ± 1 °C) and humidity (57 ± 3%) conditions, as previously reported [56,57]. All experiments were performed according to EU Directive 2010/63/EU, the Institutional Animal Care and Use Committees of Catania, and the Italian Ministry of Health (authorization n.110/2019 PR).

### 4.2. Intestinal Organoid Isolation and Culture

Intestinal organoids were obtained from the small intestine as previously reported [58]. Briefly, mouse small intestine was opened longitudinally and rinsed with cold PBS (Sigma-Aldrich, Saint Louis, MO, USA). The tissue was chopped into 5–10 mm pieces and incubated with ice-cold 30 mM EDTA to separate single cells from crypts. Pellet crypts were resuspended in Matrigel (Corning, New York, NY, USA). After Matrigel polymerization, 500 μL of the intestinal organoid culture medium (Thermo Fisher Scientific, Rodano, MI, Italy) was added. The medium was changed three times per week. After 7–10 days, organoid cultures were passaged. All experiments were performed after at least two passages.

### 4.3. Chronic Exposure to Palmitate

Palmitate (Sigma-Aldrich, Saint Louis, MO, USA) was prepared as previously reported [59] and was diluted in culture medium. To identify the best biological response to the treatment, intestinal organoids were treated with increasing palmitate concentrations (0.25, 0.50 and 1.00 mM) for different time points (24, 48 and 72 h).

### 4.4. MTT Assay

To induce lipotoxic metabolic perturbation, intestinal organoids were exposed to increasing concentrations of palmitate (from 0.25 to 1 mM) for 24, 48, and 72 h, and cell viability was analyzed through the MTT assay. The same number of organoids, isolated from three mice, was seeded in 96-well plates in two biological replicates. After 3–4 days of culture, the organoids were exposed to increasing palmitate concentrations (0.25, 0.50 and 1.00 mM) for extended periods (24, 48, and 72 h). At the end of treatment, the MTT (Sigma-Aldrich, Saint Louis, MO, USA) assay was carried out as previously reported [60].

### 4.5. Analysis of Mouse Small Intestinal Organoid Growth Treated with Palmitate

Organoid growth and lipotoxicity effect on the development of organoids were analyzed by collecting brightfield images of controls and palmitate-exposed (0.5 mM palmitate) organoids, respectively, with inverted fluorescence microscopy TI-E (Nikon, Amsterdam, The Netherlands, Europe) using a Mc DS-Qi2 Mono Digital Camera as previously reported [16]. The Nis Element AR Software (Nikon) was used to measure the following organoid parameters: the ratio between the lumen and cell length, number of buds, and crypt and villus domains. These experiments were performed by using organoids isolated from six mice.

### 4.6. Total RNA Extraction, Reverse Transcription and Quantitative PCR

After chronic exposure to 0.5 mM palmitate, organoids were harvested by dissolving Matrigel with cold PBS. Total RNA was isolated from organoids through the RNeasy Mini Kit (Qiagen) following the manufacturer’s instructions. Total RNA was stored at −80 °C. RNA concentration was determined by spectrophotometric analysis (Nanodrop, Thermo Fisher Scientific, Rodano, MI, Italy). We performed quantitative real-time PCR by using Power SYBR^®^ Green RNA-to-CT™ 1-Step Kit (Thermo Fisher Scientific, Rodano, MI, Italy) as previously reported [61,62]; beta-2-microglobulin (B2M) was used as the endogenous control gene. Gene expression dysregulation was determined through the 2−ΔΔCt method [63]. Quantitative real-time PCR was conducted on three biological replicates from five mice.

### 4.7. Immunofluorescence

Immunostaining of organoids was performed as previously reported [16]. The following antibodies were used for immunofluorescence staining: anti-FABP2 (R&D System, Abingdon, UK), anti-LGR5, anti-CCK and anti-MUC2 (Thermo Fisher Scientific, Rodano, MI, Italy). Immunofluorescence images were acquired on an inverted microscope TI-E (Nikon, Amsterdam, The Netherlands) and analyzed using Nis Element AR Software (Nikon). Immunofluorescence data were obtained by using organoids isolated from six mice, and slides were stored at −20 °C.

### 4.8. Elisa Assay

To study CCK and MUC2 production, organoids were isolated from three mice and cultured with or without 0.5 mM palmitate for 48 h in three biological replicates. After treatment, the supernatant was centrifuged and stored at −20 °C for subsequent analysis. The CCK levels in the supernatant were measured using the CCK EIA kit (Sigma-Aldrich, Saint Louis, MO, USA) according to the manufacturer’s instructions. The MUC2 levels were measured through a specific ELISA kit (Aviva Systems Biology, Clinisciences, Nanterre, France) following the manufacturer’s instructions. CCK and MUC2 levels were normalized with respect to the total number of organoids.

### 4.9. Statistical Analysis

Data are reported as means ± SD. One-way ANOVA followed by post hoc analysis for significance (Bonferroni test) was performed to determine statistical significance between different conditions. The data collected from the two groups were analyzed through Student’s t test. We considered statistically significant a *p* value lower than 0.05. GraphPad Prism version 6.0 (GraphPad Software, Inc., San Diego, CA, USA) was used for the statistical analysis.

## Data Availability

Not applicable.

## Supplementary Materials

The following supporting information can be downloaded at: https://www.mdpi.com/article/10.3390/ijms23147751/s1.
