# Supplementary material for: Molecular Effects of Chronic Exposure to Palmitate in Intestinal Organoids: A New Model to Study Obesity and Diabetes
Source: Int J Mol Sci. 2022 Jul 13;23(14):7751. doi: 10.3390/ijms23147751 (PMC9320247; doi:10.3390/ijms23147751)
Supplement: Supplementary file 1 [file ijms-23-07751-s001.zip › ijms-1807642-supplementary.pdf]

Supplementary Material

Table S1. Statistical p-values of MTT assay data.

Time points	p-value<br>C vs P 0.25mM	p-value<br>C vs P 0.5mM	p-value<br>C vs P 1 mM
24 h	0.9937	0.7512	< 0.0001
48 h	0.7249	0.0418	< 0.0001
72 h	< 0.0001	< 0.0001	< 0.0001

Table S2. Statistical p-values of organoid morphological parameters.

Morphological parameters	p-value<br>24 h	p-value<br>48 h
Number of buds	0.7298	0.0403
Crypt domain length	0.7579	0.0412
Villus domain length	0.2103	0.0243
Lumen-cell length ratio	0.1920	0.0051

Table S3. Statistical p-values of analyzed mRNA expression levels.

Gene	p-value
Lgr5	<0.0001
Prom1	<0.0001
Bmi1	0.8867
Atoh1	0.0004
Hes1	<0.0001
Ngn3	0.0047
Pou2f3	0.0514
Kfl4	0.1707
Sox9	0.9870
Fabp2	<0.0001
Chga	<0.0001
Muc2	<0.0001
Lyz1	0.5278
Cck	<0.0001
Gip	0.3642
Gcg	0.1177
Sst	0.0656
Nts	0.5721
Ghrl	0.2235
Sct	0.1160
Gast	0.0943

Table S4. Statistical p-values of immunofluorescence analysis.

Markers	Fluorescence intensity<br>p-value	Marker-cell numbers<br>p-value
CCK	0.0091	0.0028
MUC2	0.0151	0.0318
FABP2	<0.0001	0.0012
LGR5	0.0003	0.0244

Table S5. Statistical p-values of secretion data.

Secreted proteins	p-value
MUC2	<0.0001
CCK	0.0143
